# The Need to See the World: Outings and Meaningful Engagement Among Assisted Living Residents With Dementia

**DOI:** 10.1093/geroni/igaa057.1596

**Published:** 2020-12-16

**Authors:** Joy Ciofi, Candace Kemp, Stephen Duong, Andrea Hill, Anna Lisa Baidoo, AprilSpring Wood, Pamela Manley, Alexis Bender

**Affiliations:** 1 Georgia State University, Atlanta, Georgia, United States; 2 Georgia State University, Atlanta, United States; 3 Georgia State University, Macon, Georgia, United States; 4 Emory University, Atlanta, Georgia, United States

## Abstract

Meaningful engagement is a promising avenue for enhancing the lives of persons with dementia. Yet, engagement can prove challenging across care settings. Here, we report on our ongoing 5-year NIA-funded study (R01AG062310) aimed at identifying best practices for promoting meaningful engagement opportunities among assisted living residents with dementia. Guided by grounded theory method, the study involves multiple modes of qualitative data collection, including: participant observation; resident record review; and in-depth interviews with residents (when possible), assisted living staff, residents’ family and friends, volunteers, and others involved in residents’ daily lives. We present analysis of data collected in 4 diverse care settings studied in-depth over a one-year period and focus on “outings” or instances when residents leave the assisted living setting. Findings show the variable nature, content, and frequency of outings and demonstrate the advantages, disadvantages, and challenges associated with taking persons living with dementia out of their living environments. Outings represent an opportunity “to get out,” “see the world,” and “not be stuck inside.” Yet, outings are not universally successful and sometimes create frustration, distress or confusion. Multiple factors intersect to influence residents’ opportunities to participate in and derive benefit from outings, including residents’ cognitive and physical function, and personal preferences, and formal and informal care partners’ knowledge, attitudes, behaviors, and resources. Care setting practices and policies, resources, size and location, and community connections also shape opportunities for and experiences with outings among residents living with dementia. We discuss the implications of our findings for research, policy, and practice.

